# Addressing Economic Insecurities Can Improve Patient‐Reported Outcomes in Lupus

**DOI:** 10.1002/acr.80000

**Published:** 2026-03-25

**Authors:** Jay Patel, Tripti Singh, Meredith Ingersoll, Shelby Gomez, Amanda Weber, Sarah E. Panzer, Sancia Ferguson, Christie M. Bartels, Shivani Garg

**Affiliations:** ^1^ School of Medicine and Public Health University of Wisconsin–Madison Madison Wisconsin USA; ^2^ Yale School of Medicine New Haven Connecticut USA

## Abstract

**Objective:**

Economic insecurities, such as food, housing, transportation, and financial challenges, are modifiable risk factors and influence patient‐reported outcomes (PROs) in systemic lupus erythematosus (SLE). We examined the following: (1) associations between economic insecurities and PROs, and (2) the impact of screening and addressing economic insecurities during SLE visits.

**Methods:**

In the Collaborative Lupus Clinics at the University of Wisconsin–Madison, patients were routinely screened for economic insecurities and met with a social worker (SW) during visits. Clinical data including PROs from the Patient‐Reported Outcomes Measurement Information System Global Health Short Form at baseline and follow‐up were abstracted from the Collaborative Lupus Clinics Data Repository. Using multivariable linear regression, associations among economic insecurities, social drivers of health (eg, insurance), and PROs were assessed. Next, changes in PROs following SW discussions were evaluated.

**Results:**

Among 222 patients (mean age 47 years; 90% women), 16% reported at least one economic insecurity. Each 1‐point increase in economic insecurity score was linked with lower PRO T scores in all domains: physical health (−1.85, *P* = 0.04), mental health (−1.32, *P* = 0.17), and social function (−0.24, *P* = 0.03). A sequential increase in economic insecurities, at least one, at least two, and at least three, lowered physical health by 2.00, 3.86, and 9.10 points, respectively. Patients with economic insecurities and Medicaid/no insurance had two times lower PRO scores in all domains. Following SW intervention, PROs improved by 4.52, 1.12, and 0.69 points in all domains.

**Conclusion:**

Although economic insecurities negatively affect PROs in SLE, a systematic approach to assess and address economic insecurities in clinics can improve PROs over time in SLE.

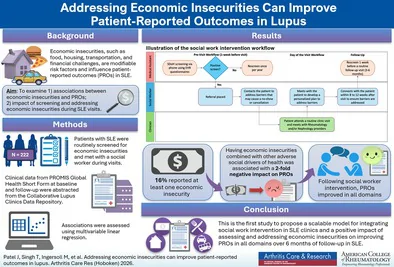

## INTRODUCTION

Systemic lupus erythematosus (SLE; or lupus) is the most common life‐threatening autoimmune disease.^1^ Individuals with lupus face a 20‐fold higher risk of end‐stage kidney disease, a six‐fold higher risk of cardiovascular disease, and a six‐fold higher risk of premature death.^2^, ^3^, ^4^, ^5^ Likewise, individuals with lupus often have lower than average scores in patient‐reported outcomes (PROs), a reflection of an individual's self‐evaluation of health due to chronic disease. Poor PROs in lupus could likely explain 31% more work disability compared to other diseases.^6^ However, biologic factors and genetic variability cannot fully account for the heterogeneity in clinic outcomes and PROs.^7^ To improve lupus outcomes, it is important to understand how social drivers of health (SDoH) impact clinical outcomes and PROs in lupus.

**SIGNIFICANCE & INNOVATIONS**

- This study validates economic insecurities as a distinct risk factor associated with worse patient‐reported outcomes (PROs) in systemic lupus erythematosus (SLE).
- A sequential negative impact on all PRO domains is seen with increasing economic insecurities in SLE, and having economic insecurities combined with other adverse social drivers of health (SDoH) is associated with a two‐fold negative impact on PROs.
- This study highlights a systematic approach to using a validated electronic health record–based screening tool (Epic SDoH Snapshot) in clinics for assessing economic insecurities.
- This is the first study to propose a scalable model for integrating social work intervention in SLE clinics and a positive impact of assessing and addressing economic insecurities on improving PROs in all domains over six months’ follow‐up in SLE.

Our group's meta‐analysis reported a multiplicative negative interaction two or more adverse SDoH driving 12‐fold higher odds of poor clinical outcomes in lupus nephritis (LN).^8^ However, similar associations among SDoH, biologic factors, and PROs have not been established across geographically different population groups in lupus. A recent study using the California Lupus (CLUES) cohort highlighted that economic insecurities (ie, food, housing, transportation, financial insecurity) and not conventionally measured SDoH (ie, employment, insurance) were associated with poor PROs.^9^ However, there are stark differences in SLE presentation, SDoH, economic insecurities, insurance coverage, and health care access in the Midwest region compared to the West.^10^, ^11^ Therefore, there is a need to validate associations between PROs and SDoH in other cohorts to enhance generalizability. Additionally, data on how SDoH should be assessed and addressed during a clinic visit are lacking. Finally, it is important to estimate the impact of addressing modifiable SDoH on PROs and clinical outcomes in lupus. Such data are critical for identifying key factors among SDoH domains, the interactions among such factors, potentially modifiable targets, and guidance on how to intervene to improve outcomes, particularly PROs, in lupus.

Therefore, leveraging data from the Collaborative Lupus Clinics’ Data Repository at the University of Wisconsin–Madison (UW),^12^ we aimed to examine associations between economic insecurities and conventional SDoH with PROs in lupus. Additionally, we tested interactions between economic insecurities and conventional SDoH and the impact of such interactions on PROs to identify high‐risk groups and high‐risk social factors. Finally, we assessed the longitudinal impact of a social worker (SW)‐based intervention, which has been integrated into our Collaborative Lupus Clinics as part of routine care,^12^ on addressing economic insecurities and improving PROs in our cohort.

## METHODS

### Collaborative Lupus Clinics teams at the UW

We have established multidisciplinary collaborative lupus and LN clinics at the UW. The UW Collaborative Lupus Clinics care for more than 400 unique patients and provide over 700 patient visits annually, serving statewide. Approximately 35% of patients identify as Asian or Black/African American. These teams comprise rheumatologists, nephrologists, an SW, skilled medical staff, and pharmacists. We have created workflows to screen and address SDoH as part of routine lupus care in our clinics. More details about the multidisciplinary clinics can be found in our 2022 manuscript.^12^

### Routine clinical workflow for SDoH screening and SW‐driven intervention in our Collaborative Lupus Clinics

**Figure 1:**
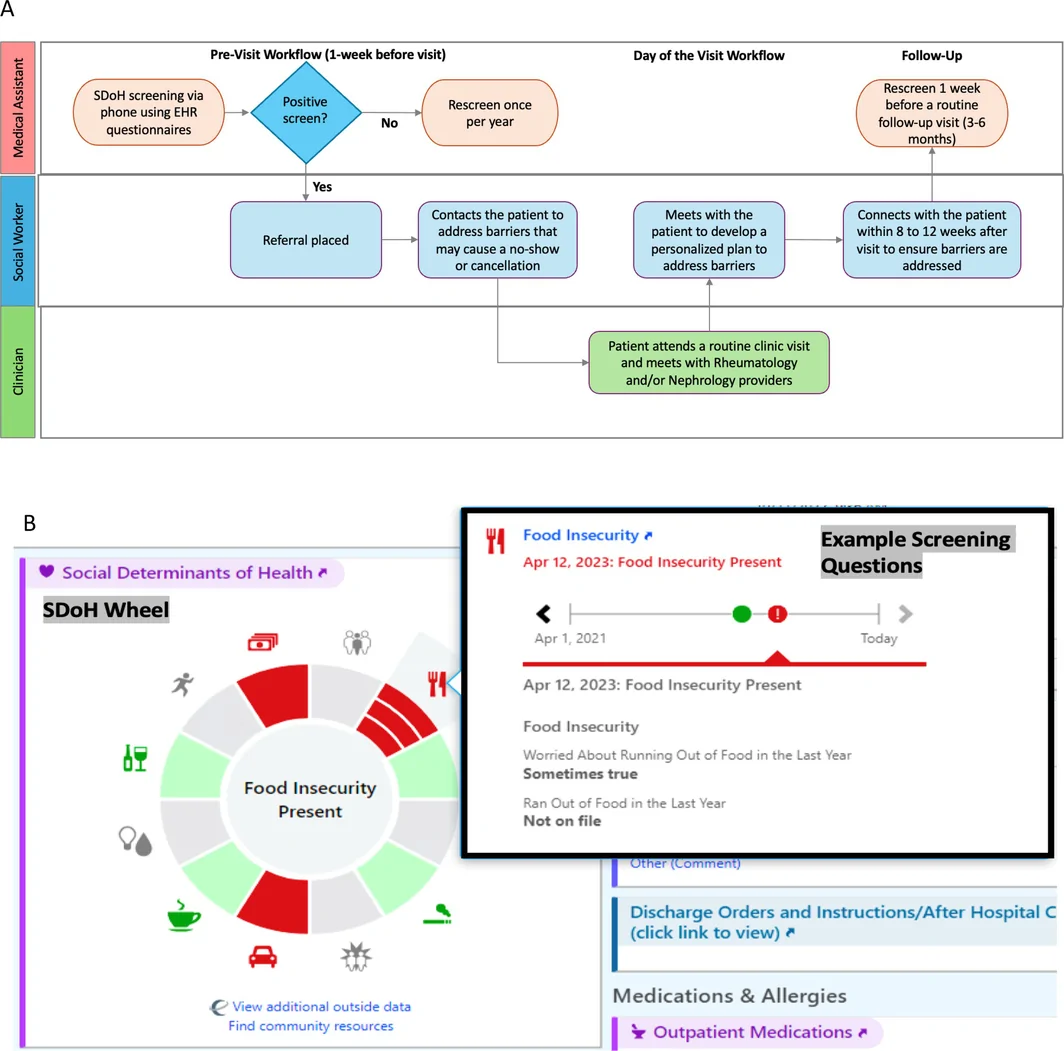
Clinical workflow to assess and address economic insecurities at baseline lupus visit. (A) Social work intervention workflow in our Collaborative Lupus Clinics at the University of Wisconsin–Madison. (B) The screening tool with the Epic SDoH Snapshot, part of Epic's Healthy Planet module, supports social risk factor screening, including food insecurity, transportation needs, and financial insecurity. EHR, electronic health record; SDoH, social drivers of health.

We developed a workflow to screen for social risk factors (including economic insecurities) as part of routine lupus care, using the Epic electronic health record (EHR). The workflow includes four steps (Figure 1A).

#### Step 1: previsit screening

One week before the visit, as part of our previsit screening, a medical assistant on the lupus clinic team calls all new and established patients with a diagnosis of lupus or LN who are scheduled to be seen in the UW's Collaborative Lupus Clinics. During the call, the medical assistant screens for social barriers that are currently present and confirms that the laboratory tests are completed before the scheduled visit. We used the existing SDoH screening tool, available within the Epic EHR and in concordance with the guidelines from the National Academy of Medicine (NAM) and Center for Medicare and Medicaid Services (CMS), to screen for economic insecurities in our clinics.^13^, ^14^ To elaborate, the SDoH screening tool that we used uses validated screening questions to identify four different economic insecurities including transportation barriers, financial barriers, food insecurities, and unstable housing. Details on validated questions used to screen for these economic insecurities are listed in Figure 1B and Supplementary Table 1. Positive screens for any social barriers were shared with our SW in UW Collaborative Lupus Clinics. The SW contacted patients with at least one social barrier to address any barriers that could otherwise result in no‐shows or cancellations (Figure 1A).

#### Step 2: during clinic visit

**Figure 2:**
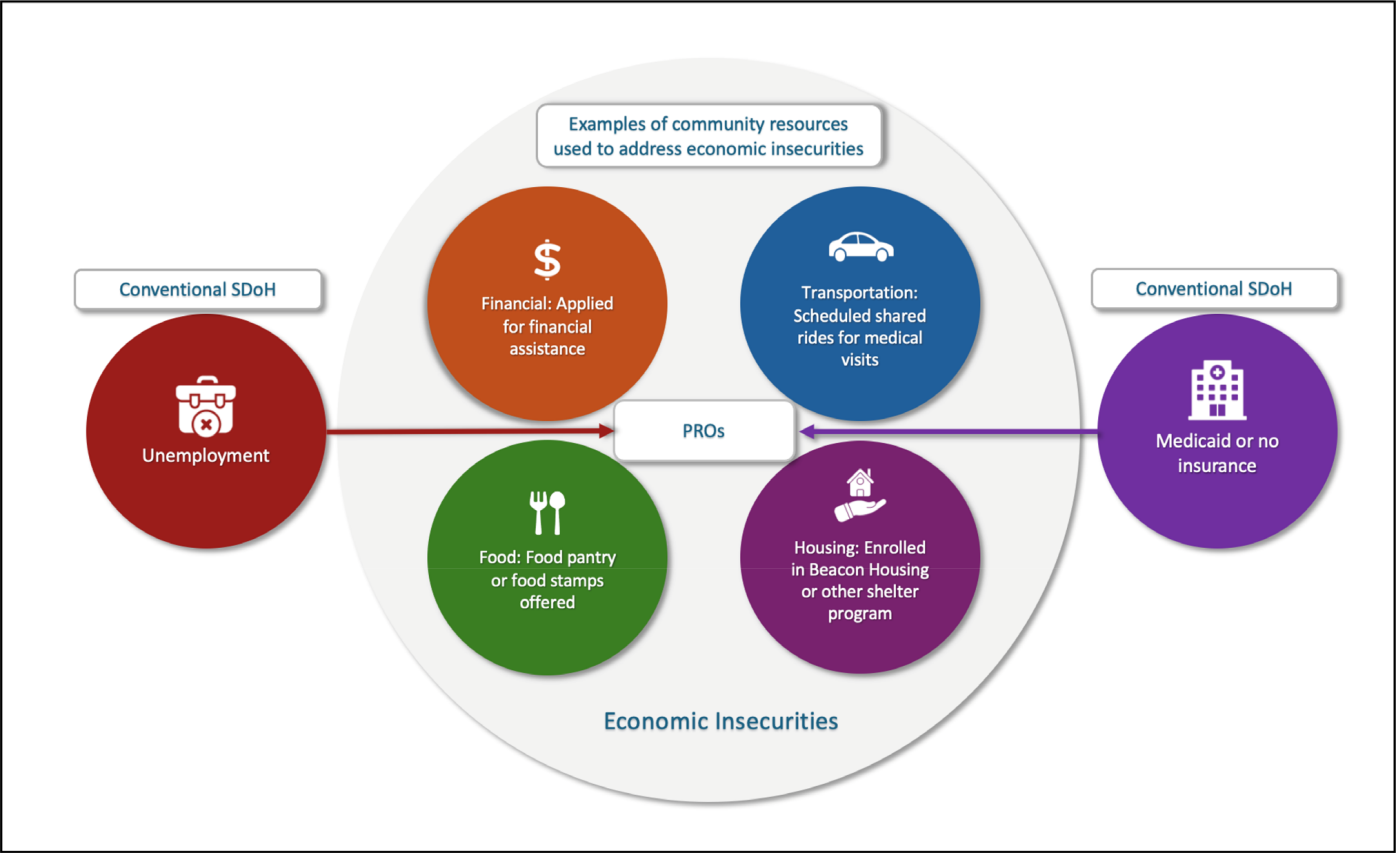
A model showing modifiable social risk factors (economic insecurities) and other social drivers of poor patient‐reported outcomes (PROs) in lupus and how economic insecurities can be addressed, leveraging community resources and social workers’ support in clinics. SDoH, social drivers of health. Color figure can be viewed in the online issue, which is available at http://onlinelibrary.wiley.com/doi/10.1002/acr.80000/abstract.

For patients with any economic insecurities, the SW met with them during their lupus clinic visit and developed a personalized plan with the patients to address their barriers. This plan included sharing resources such as providing food stamps, setting up rides for medical visits, applying for financial assistance, or connecting with the pharmacist for copay or medicine coverage issues (Figure 2).

#### Step 3: after clinic visit follow‐up

The SW connected with the patient via phone or a message within 8 to 12 weeks after the visit to ensure that the barriers were addressed or resolved. If economic insecurities were resolved, then the EHR questionnaire was updated.

#### Step 4: rescreening for SDoH

For patients who screened positive for any social barriers or economic insecurities in the last three to six months, they were rescreened one week before their three‐ to six‐month follow‐up visit as part of previsit screening (step 1). Patients who had never reported any social barriers were only screened once a year in our clinics.

### Study design

This is a medical records review observational study that used data from the Collaborative Lupus Clinics’ Data Repository at the UW in 2020 to 2024. The participants in this Collaborative Lupus Clinics’ Data Repository met the following eligibility criteria: (1) residency in Wisconsin, Illinois, or neighboring states; (2) age ≥18 years; (3) the ability to provide informed consent for the study team to access medical records for data abstraction over time; and (4) a validated diagnosis of SLE according to the 1997 American College of Rheumatology (ACR) SLE classification criteria,^15^ 2012 Systemic Lupus International Clinics (SLICC) classification criteria,^16^ or 2019 EULAR/ACR classification for SLE.^17^ The study protocol was approved by the UW School of Medicine and Public Health Institutional Review Board (IRB; 2019‐0942).

### Data collection

At every lupus clinic visit, the following data were completed as part of clinical care: the Patient‐Reported Outcomes Measurement Information System (PROMIS) Global Health Short Form completed by patients as a PRO measure,^18^ patient‐reported medication adherence, and clinician‐reported SLE Disease Activity Index 2000 (SLEDAI‐2K)^19^ and SLICC Damage Index (SDI).^20^ Data were manually abstracted by a trained research coordinator for a priori defined clinical variables (IRB 2019‐0942) from each consented patient's EHR every quarter. Periodic reviews were conducted by the study team to ensure data reliability and completeness.

### Variables

#### Key explanatory variable: economic insecurities

From the index visit date, we abstracted data on economic insecurities, including housing instability, food insecurity, financial insecurity (eg, difficulty affording utilities, medical care, housing), and transportation barriers (eg, missing medical appointments or difficulty obtaining medications). Given a person could experience multiple economic insecurities, we used economic insecurities as a continuous variable in which each additional economic insecurity was represented as a 1‐unit increase.^9^ In a separate model, economic insecurities were categorized as at least one, at least two, and at least three economic insecurities being present.

#### Other variables: conventional SDoH

From the index visit date, we also abstracted data on conventional SDoH domains including individual, health behaviors, and community. These data included employment status, which was categorized as unemployed, employed, student, or retired. Next, insurance was categorized as Medicaid/no insurance versus commercial or private insurance. Medicaid eligibility or uninsured status grouping served as a surrogate for low personal income. For community SDoH domain, we calculated Area Deprivation Index (ADI) and categorized it as <80% decile versus ≥80% decile, indicating neighborhoods at highest risk of disadvantage. We also abstracted data on high‐risk behaviors, (smoking, recreational drug use, alcohol use, etc). Finally, given mental health factors such as patient‐reported loneliness or depression could affect PROs, this information was also abstracted.^8^

### Covariables: sociodemographic and other factors

From the index clinic visit, we abstracted key demographics and SLE‐specific variables from the participants’ medical records. Demographics included age (per 10‐year increase), patient‐reported sex (male vs female), and race or ethnicity (White, Black or African American, Hispanic or Latino, Asian). SLE‐specific variables included SLEDAI‐2K as a continuous variable (per 1‐unit increase),^19^ SDI as a continuous variable (1‐unit increase),^20^ and duration of SLE in years. Next, we abstracted data on immunosuppressive medications, prednisone dose at the visit in milligrams (per 1‐mg increase), and hydroxychloroquine (HCQ) whole blood (WB) levels (>750 vs ≤750 ng/mL) as a proxy for effective HCQ use.^21^, ^22^, ^23^

### Primary outcome: PROs

From the index clinic visit and three‐ to six‐month follow‐up clinic visit, PROs of physical health, mental health, and social function were collected from the PROMIS Global Health Short Form (10 questions).^18^ Raw scores for PROs for physical health, mental health, and social function were calculated and converted to normalized T scores using the standard validated PROMIS scoring guidelines.^24^ Normalized T scores for PROs for physical health, mental health, and social function were used as continuous variables.

### Statistical methods

#### Cross‐sectional analysis using data from index clinic visit

We first performed descriptive analysis and summarized the baseline characteristics of our cohort using means and standard deviations for continuous variables and frequencies for categorical variables.

Next, unadjusted linear regression analysis was completed to understand the association between each variable, including demographics, SLE‐specific variables, medications, conventional SDoH, and economic insecurities, with PRO T scores for physical health, mental health, and social function. PRO T scores for physical health, mental health, and social function were analyzed in separate models to understand how different factors affect PROs in each domain.

Then, multivariate linear regression analyses were performed to examine associations among the key explanatory variable, economic insecurities, and PRO T scores in physical health, mental health, and social function adjusting for covariates. Again, separate models were built to analyze associations or drivers of PRO T scores in each domain. Our multivariable models were adjusted for age, sex, race or ethnic groups, SLEDAI score, SDI score, prednisone dose, ADI score, HCQ WB levels, high‐risk health behavior, and patient‐reported loneliness or depression. Each PRO (physical health, mental health, and social function) was modeled as a separate continuous outcome variable, and results were reported as mean difference or change in mean.

Additionally, to understand the sequential impact of increasing economic insecurities on PROs, we used economic insecurities as a categorical variable and the following categories were used in the analysis: at least one economic insecurity, at least two economic insecurities, and at least three economic insecurities. For each category of economic insecurity, we performed multivariable linear regression analysis to calculate the mean change in PRO in each domain (physical health, mental health, social function) with versus without economic insecurities. To assess the linear trend across increasing levels of economic insecurity on each domain, we performed a *P* for trend analysis.

Finally, we performed three subgroup analyses to test interactions between economic insecurities and conventional SDoH (insurance status, Medicaid/no insurance vs commercial insurance; employment status, unemployed; race, Black or African American). These groups were selected due to their established association with worse clinical outcomes.^8^ Given limited sample size, reduced multivariable regression models were developed to assess how economic insecurities resulted in mean change in PRO in each subgroup, adjusting for only key covariables.

#### Longitudinal analysis comparing change in PROs at three‐ to six‐month follow‐up visit to baseline visit

Using a pre–post multivariable linear regression model, we assessed if addressing or resolving economic insecurities with the help of community resources or SW resulted in a change in PROs in each domain. We included both unadjusted and adjusted models. Variables adjusted for were age, race, sex, insurance status, ADI score, SLEDAI score at the follow‐up visit, SDI score at the follow‐up visit, prednisone dose, HCQ WB levels, and medications at the follow‐up visit. Economic insecurities addressed (yes or no) was the key explanatory variable in this model.

#### Missing data handling

We imputed missing variables using median values derived from participants’ clinic visits within six months before the index visit. We performed a sensitivity analysis in which we first excluded participants with missing outcome variables (PRO scores) and then used imputed data. Given similar results and only missing PROs from five visits, we used the imputed data for the final models to avoid sample size reduction.

Given an observational cohort, we did not calculate a sample size. Using power.t.test in R software, we estimated that a sample size of 222 would detect even a small change of 0.22 in mean PRO T scores given the power was set at 90% and a two‐sided alpha set at 0.05. All analyses were conducted using R software (version 2023.12.1). Statistical significance was defined as *P* < 0.05, and all results were reported with 95% confidence intervals (CIs).

## RESULTS

This study included 222 participants (mean ± SD age 46.5 ± 16.9 years), of whom 90% (n = 199) were female. Racial and ethnic distribution was 73% White (n = 163), 13% Black or African American (n = 28), 9% Asian (n = 19), and 5% Hispanic or Latino (n = 12). At baseline, conventional SDoH included 31 patients with Medicaid or no insurance (14%), and 46 patients were unemployed (21%). A total of 36 patients reported at least one economic insecurity including transportation barriers, housing instability, food insecurity, or financial insecurity (16%) during their baseline visit (Table 1). The mean SLEDAI score was 2.8 ± 3.3; SDI score was 0.89 ± 1.1. Mean T scores were as follows: physical health 43.8 ± 7.45, mental health 45.5 ± 7.87, and social function 3.10 ± 0.89. Patients with at least economic insecurity had lower T scores across all domains: physical health 40.7 ± 6.88, mental health 42.3 ± 7.94, and social function 2.72 ± 0.81. Other patient characteristics at baseline are shown in Table 1.

**Table 1:** Baseline characteristics (n = 222 patients)*

Variables at clinic visit	Mean ± SD or n (%)
Age at enrollment, y	46.5 ± 16.9
Female	199 (90)
Race and ethnicity (patient reported)	
White	163 (73)
Black or African American	28 (13)
Asian	19 (9)
Hispanic or Latino	12 (5)
Insurance type	
Commercial or Medicare	191 (86)
No insurance or Medicaid	31 (14)
Employment status	
Employed	136 (61)
Unemployed	46 (21)
Retired	32 (14)
Student	8 (4)
Area Deprivation Index < 80th percentile	17 (8)
Body weight, kg	80 ± 21
SLE disease duration, y	5.6 ± 4.9
Glomerular filtration rate, mL/min/1.73 m^2^	88 ± 27
Patient‐reported loneliness or depression	14 (6)
High‐risk health behavior	9 (4)
Economic insecurities	
None	186 (84)
At least one	36 (16)
At least two	11 (5)
Three or more	4 (2)
SLEDAI‐2K score	2.8 ± 3.3
SDI score	0.89 ± 1.1
HCQ dose, mg	323 ± 83
Weight‐based HCQ dose ≤5 mg/kg/d	186 (84)
Therapeutic HCQ whole blood levels >750 ng/mL	123 (55)
Other SLE medicines	
Disease‐modifying agents	69 (31)
Biologics	15 (7)
Combination therapy	13 (6)
Prednisone use	69 (31)
Prednisone dose, mg	3.2 ± 9.0

* HCQ, hydroxychloroquine; SDI, Systemic Lupus International Collaborating Clinics Damage Index; SLEDAI‐2K, Systemic Lupus Erythematosus Disease Activity Index 2000.

### Associations between economic insecurities and PROs using baseline visit data

Unadjusted bivariate analyses showed that each additional economic insecurity was associated with lower PRO T scores in physical health (−2.72, 95% CI −4.23 to −1.22, *P* < 0.001), mental health (−2.62, 95% CI −4.22 to −1.03, *P* = 0.001), and social function (−0.34, 95% CI −0.53 to −0.17, *P* = 0.0002) domains, respectively (Supplementary Table 2). After adjusting for covariables, each additional economic insecurity was independently associated with a 1.85‐point lower PRO T score in the physical health domain (95% CI −3.58 to −0.11, *P* = 0.04; Table 2) and 0.24‐point lower PRO T score in the social function domain (95% CI −0.45 to −0.03, *P* = 0.03; Table 2). However, no significant associations were noted between the presence of economic insecurity and PRO T score in the mental health domain (mean difference −1.32, 95% CI −3.20 to 0.56, *P* = 0.17; Table 2) after controlling for all factors. Other variables independently associated with lower PROs T scores in adjusted models included unemployment (associated with 3.09 and 0.49 lower PRO T scores in physical and social health domains; Table 2) and being Black or African American (associated with 3.33 and 3.65 lower PRO T scores in physical and mental health domains; Table 2).

**Table 2:** Multivariable linear regression showing adjusted associations among economic insecurities, conventional SDoH, and PROs (n = 222)*

Variables at visit	Physical health		Mental health		Social function	
	Adjusted mean change (95% CI)	*P* value	Adjusted mean change (95% CI)	*P* value	Adjusted mean change (95% CI)	*P* value
Age per 10‐year increase	0.018 (−0.06 to 0.09)	0.64	0.05 (−0.02 to 0.14)	0.15	−0.0003 (−0.01 to 0.01)	0.94
Female	0.16 (−3.27 to 3.58)	0.92	−1.31 (−5.03 to 2.41)	0.49	−0.20 (−0.62 to 0.21)	0.34
Black or African American	**−3.33 (−0.24 to −6.43)**	**0.03**	**−3.65 (−0.29 to −7.01)**	**0.03**	−0.18 (−0.55 to 0.20)	0.36
Asian	1.37 (−2.29 to 5.03)	0.46	0.45 (−3.52 to 4.43)	0.82	0.04 (−0.41 to 0.48)	0.87
Hispanic or Latino	1.23 (−3.25 to 5.71)	0.59	1.28 (−3.58 to 6.14)	0.60	0.21 (−0.33 to 0.76)	0.44
Body weight per 1‐kg increase	−0.04 (−0.09 to 0.01)	0.09	−0.02 (−0.07 to 0.03)	0.65	−0.002 (−0.01 to 0.004)	0.58
Medicaid insurance/no insurance	−0.87 (−3.90 to 2.17)	0.57	−1.35 (−4.65 to 1.94)	0.42	−0.18 (−0.54 to 0.19)	0.35
ADI <80% decile	−1.35 (−5.06 to 2.37)	0.48	−0.92 (−4.96 to 3.11)	0.65	−0.11 (−0.56 to 0.34)	0.63
Loneliness or depression	2.36 (−2.00 to 6.72)	0.29	−1.65 (−6.39 to 3.08)	0.49	0.16 (−0.37 to 0.69)	0.55
High‐risk health behavior per 1‐unit increase	−0.56 (−5.69 to 4.57)	0.83	−2.30 (−7.87 to 3.27)	0.42	−0.13 (−0.75 to 0.49)	0.68
Employment						
Unemployed	**−3.09 (−0.31 to −5.87)**	**0.03**	−2.55 (−5.56 to 0.47)	0.10	**−0.49 (−0.16 to −0.83)**	**0.004**
Retired	−2.01 (−5.40 to 1.39)	0.25	0.42 (−3.27 to 4.10)	0.82	0.14 (−0.27 to 0.56)	0.49
Student	−0.99 (−6.90 to 4.92)	0.74	1.75 (−4.67 to 8.17)	0.59	−0.05 (−0.80 to 0.67)	0.89
Economic insecurities per 1‐unit increase	**−1.85 (−0.11 to −3.58)**	**0.04**	−1.32 (−3.20 to 0.56)	0.17	**−0.24 (−0.03 to −0.45)**	**0.03**
SLEDAI score per 1‐unit increase	−0.11 (−0.45 to 0.24)	0.55	0.03 (−0.35 to 0.41)	0.88	−0.004 (−0.05 to 0.04)	0.87
SDI score per 1‐unit increase	−0.81 (−1.78 to 0.16)	0.10	−0.29 (−1.34 to 0.76)	0.59	−0.04 (−0.16 to 0.08)	0.51
Lupus duration per one‐year increase	−0.01 (−0.20 to 0.21)	0.94	0.06 (−0.16 to 0.28)	0.59	0.003 (−0.02 to 0.03)	0.80
Hydroxychloroquine blood levels > 750ng/mL	−0.59 (−2.68 to 1.50)	0.58	0.52 (−1.75 to 2.79)	0.65	−0.05 (−0.30 to 0.21)	0.72
Prednisone dose at visit per 1‐mg increase	−0.07 (−0.18 to 0.05)	0.25	−0.03 (−0.16 to 0.10)	0.62	−0.01 (−0.02 to 0.003)	0.13
Immunosuppression	−0.94 (−2.24 to 0.35)	0.15	0.01 (−1.40 to 1.42)	0.99	−0.07 (−0.23 to 0.09)	0.37

* Data are mean change in PROs (95% CI, with *P* < 0.05 indicating significance). Lower scores indicate worse PROs. ADI, Area Deprivation Index; CI, confidence interval; PRO, patient‐reported outcome; SDI, Systemic Lupus International Collaborating Clinics Damage Index; SDoH, social drivers of health; SLEDAI, Systemic Lupus Erythematosus Disease Activity Index. Bold font indicates significant *p*‐value <0.05.

### Sequential negative impact of increasing economic insecurities

Next, across economic insecurity categories (at least one, at least two, and at least three), a sequential increase in the negative impact of multiple economic insecurities on PROs was noted, particularly in the physical health and social function domains (Supplementary Table 3). To elaborate, patients reporting at least one, at least two, and at least three insecurities had a sequential increase in the mean difference of −2.0, −3.9, and −9.1, respectively, in PRO T scores in the physical health domain (Supplementary Table 3) and −0.3, −0.4, and −1.3, respectively, in the social function domain (Supplementary Table 3). A similar trend showed −1.8, −2.6, and −5.8, respectively, lower T scores in the mental health domain across economic insecurity categories, but these were not statistically significant (*P* > 0.05; Supplementary Table 3), adjusting for covariables including depression and loneliness (Supplementary Table 3). Our unadjusted models revealed a linear association between increasing economic insecurities and lower patient‐reported physical health (*P* = 0.0005; Supplementary Figure 1A), mental health (*P* = 0.001; Supplementary Figure 1B), and social function (*P* = 0.0002; Supplementary Figure 1C).

### Subgroup analyses

To further understand interactions between conventional SDoH and economic insecurities, we performed subgroup analyses. In the first subgroup analysis, we only included patients with Medicaid or no insurance. We noted that having economic insecurities in this subgroup had a significant multiplicative negative impact on PROs in all domains (a 4.38‐point lower T score in physical health, a 6.47‐point lower T score in mental health, and a 0.58‐point lower T score in social function; Table 3). This observed change in PRO T scores was higher than the expected additive change of mean PRO T scores (observed −4.38 > expected −2.72 [−1.85 + −0.87]).

**Table 3:** Variable impact of economic insecurities on patient‐reported physical health, mental health, and social function within (1) Medicaid/no insurance, (2) unemployment, (3) Black or African American subgroups*

Variables at clinic visit	Physical health		Mental health		Social function	
	Adjusted mean difference (95% CI)	*P* value	Adjusted mean difference (95% CI)	*P* value	Adjusted mean difference (95% CI)	*P* value
Medicaid/no insurance subgroup (n = 31)						
Economic insecurities per 1‐unit increase	**−4.38 (−0.62 to −8.13)**	**0.02**	**−6.47 (−1.94 to −11.00)**	**0.01**	**−0.58 (−0.06 to −1.11)**	**0.03**
Age per 10‐year increase	0.09 (−0.17 to 0.34)	0.50	0.26 (−0.05 to 0.57)	0.09	0.01 (−0.03 to 0.04)	0.67
Female	3.42 (−6.21 to 13.00)	0.47	−5.69 (−17.30 to 5.95)	0.32	0.33 (−1.02 to 1.68)	0.62
Black or African American	−3.43 (−10.80 to 3.90)	0.34	−1.11 (−9.97 to 7.74)	0.80	0.28 (−0.75 to 1.30)	0.58
Asian	10.00 (−2.62 to 22.70)	0.12	5.26 (−10 to 20.5)	0.48	0.92 (−0.86 to 2.69)	0.30
Hispanic or Latino	4.91 (−6.97 to 16.80)	0.40	10.40 (−3.96 to 24.70)	0.15	0.33 (−1.33 to 1.99)	0.69
SDI score per 1‐unit increase	−3.11 (−7.65 to 1.44)	0.17	−4.59 (−10.10 to 0.90)	0.10	−0.20 (−0.83 to 0.44)	0.53
Unemployed or student subgroup (n = 54)						
Economic insecurities per 1‐unit increase	**−2.18 (−0.14 to −4.22)**	**0.04**	−1.85 (−3.95 to 0.25)	0.25	**−0.29 (−0.05 to −0.53)**	**0.02**
Age per 10‐year increase	−0.03 (−0.17 to 0.10)	0.64	−0.04 (−0.18 to 0.10)	0.58	−0.01 (−0.02 to 0.01)	0.28
Female	2.01 (−4.36 to 8.37)	0.53	−6.29 (12.80 to −0.26)	0.06	0.01 (−0.73 to 0.75)	0.98
Black or African American	1.08 (−2.94 to 5.10)	0.59	0.53 (−3.61 to 4.66)	0.80	0.16 (−0.31 to 0.63)	0.49
Medicaid insurance/no insurance	−4.08 (−8.34 to 0.19)	0.06	**−5.45 (−1.06 to −9.84)**	**0.02**	**−0.50 (−0.001 to −1.00)**	**0.05**
SDI score per 1‐unit increase	−0.55 (−1.93 to 0.84)	0.43	0.12 (−1.30 to 1.55)	0.86	0.06 (−0.10 to 0.23)	0.43
Black or African American subgroup (n = 28)						
Economic insecurities per 1‐unit increase	−2.04 (−4.82 to 0.74)	0.15	−1.28 (−4.25 to 1.68)	0.40	−0.08 (−0.42 to 0.25)	0.63
Age per 10‐year increase	0.03 (−0.04 to 0.10)	0.35	**0.09 (0.02 to 0.17)**	**0.01**	0.01 (−0.002 to 0.01)	0.13
Female	1.31 (−2.45 to 5.07)	0.49	0.25 (−3.76 to 4.25)	0.90	−0.10 (−0.56 to 0.35)	0.65
Medicaid insurance/no insurance	−1.42 (−5.03 to 2.19)	0.44	−1.99 (−5.84 to 1.86)	0.10	−0.42 (−0.86 to 0.01)	0.054
SDI score per 1‐unit increase	**−1.24 (−0.11 to −2.36)**	**0.03**	−0.53 (−1.72 to 0.67)	0.39	**−0.17 (−0.03 to −0.30)**	**0.02**

* Data are adjusted mean differences (95% CI, *P* < 0.05 indicating significance) in PROs associated with a one‐unit increase in economic insecurity within each subgroup (Medicaid/no insurance, unemployment, and Black or African American) compared to no economic insecurity. Adjusted covariates are included. Lower scores indicate worse PROs. CI, confidence interval; PRO, patient‐reported outcome; SDI, Systemic Lupus International Collaborating Clinics Damage Index. Bold font indicates significant *p*‐value <0.05.

The next subgroup included only unemployed participants. Again, having economic insecurities had an adverse impact on PROs in physical and social domains (mean difference in physical health PRO −2.18, 95% CI −4.22 to −0.14, *P* = 0.04; social function PRO −0.29, 95% CI −0.53 to −0.05, *P* = 0.02; Table 3). Finally, in a Black or African American subgroup, economic insecurities were not significantly associated with PROs after controlling for other factors including age, sex, insurance type, and SDI score (Table 3).

### Longitudinal impact of assessing and addressing economic insecurities during clinic visits on PROs over time

**Figure 3:**
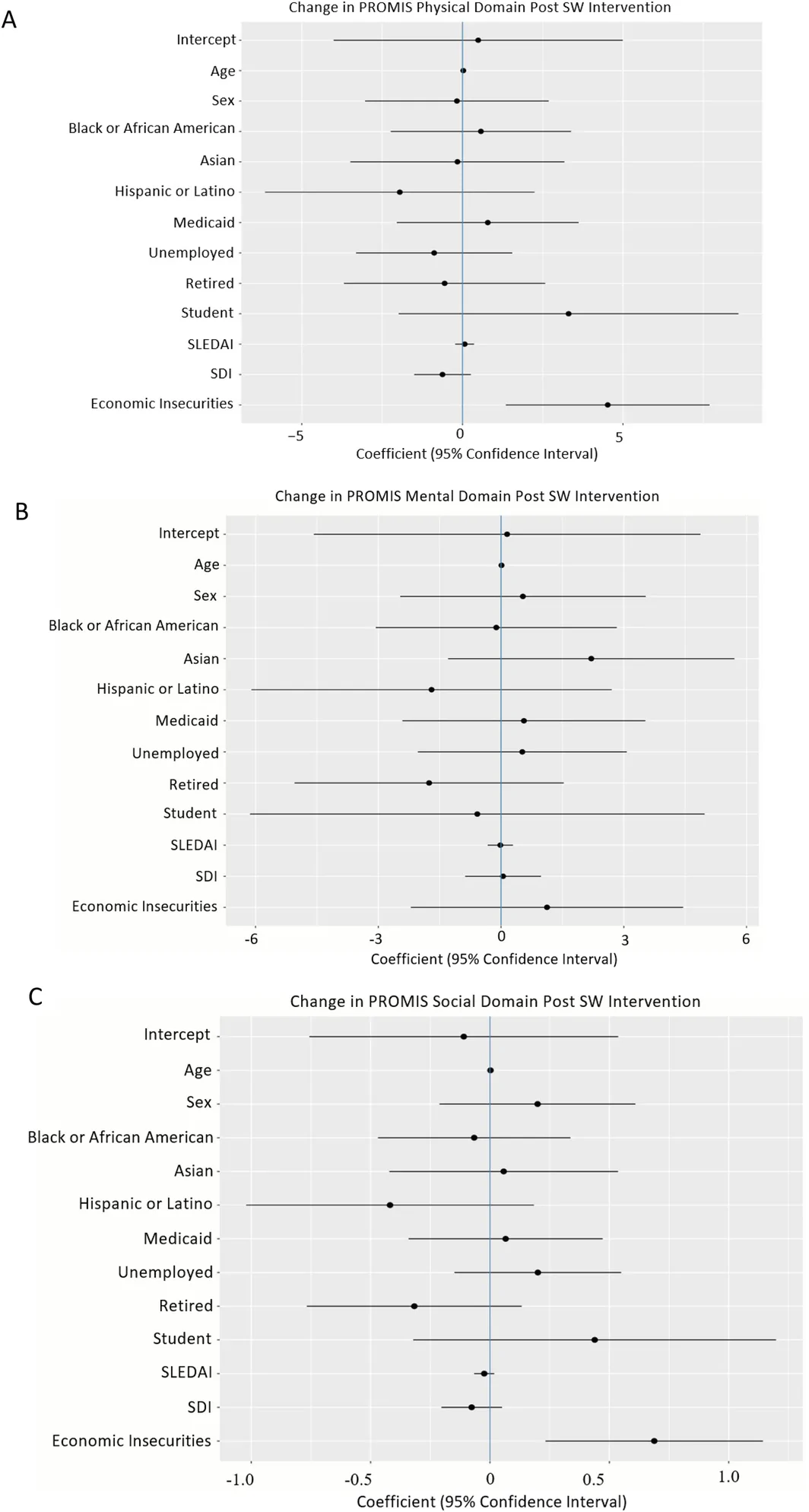
Adjusted mean change in patient‐reported (A) physical health, (B) mental health, and (C) social function after social work intervention compared to baseline scores. Points are adjusted means, and error bars are 95% confidence intervals. Data were calculated using pre–post multivariable linear regression analysis adjusting for age, sex, race, ethnicity, Medicaid insurance, employment status, SLEDAI, and SDI. PROMIS, Patient‐Reported Outcomes Measurement Information System; SDI, Systemic Lupus International Collaborating Clinics Damage Index; SLEDAI, Systemic Lupus Erythematosus Disease Activity Index; SW, social worker. Color figure can be viewed in the online issue, which is available at http://onlinelibrary.wiley.com/doi/10.1002/acr.80000/abstract.

We used data on PROs at the index clinic visit and then the three‐ to six‐month follow‐up visit (after meeting with an SW if indicated based on the presence of one or more economic insecurities). Among 222 total patients, we identified 36 patients with at least one economic insecurity who were referred to an SW or received community support. Among 36 patients, 22 patients (61%) reported resolution of economic barriers at their three‐ to six‐month follow‐up. Using an unadjusted pre–post model in this subgroup, PRO T scores significantly improved in the physical health and social function domains by 5.25 (95% CI 1.59–8.91, *P* = 0.007; Supplementary Table 4) and 0.65 (95% CI 0.09–1.21, *P* = 0.03; Supplementary Table 4) compared to baseline PRO scores. Using an adjusted linear regression analysis adjusted for covariables, we noted a significant improvement in PRO T scores in the physical health and social function domains by 4.52 (95% CI 1.35–7.69, *P* = 0.005; Figure 3) and 0.69 (95% CI 0.23–1.14, *P* = 0.003; Figure 3) postintervention compared to the baseline scores. Mean change in PRO T scores in the mental health domain were not statistically significant for both unadjusted (2.41, 95% CI −1.84 to 6.66, *P* = 0.25; Supplementary Table 4) and adjusted (1.12, 95% CI −2.21 to 4.45, *P* = 0.51; Figure 3) models.

## DISCUSSION

There is a potential interplay between SDoH and biologic factors, driving poor and heterogeneous SLE outcomes. However, prospective impacts on PROs need to be further elucidated across different populations, particularly identifying and addressing primary nonbiologic (eg, SDoH) drivers of poor outcomes in SLE. Our study directly addresses these gaps by highlighting the following: (1) economic insecurities, such as transportation barriers, financial insecurity, housing instability, and food insecurity, are associated with worse PROs consistent with recent studies^9^, ^13^; (2) a sequential negative impact of increasing economic insecurities on PROs; (3) a systematic and successful approach to screen and address economic insecurities in clinics; and (4) the positive impact of addressing economic insecurities in clinics on PROs, reinforcing their role as modifiable risk factors.

Yelin et al highlighted patients’ perspectives on how economic insecurities drove SLE flares and poor physical and mental health.^25^ A patient from the study by Yelin et al said, “Money makes it [SLE] worse… I worry about money… and paying my mortgage…”^25^, ^26^ Several studies reported that conventional SDoH, such as education, income, poverty, and ADI score, have a negative impact on PROs.^27^, ^28^, ^29^ The CLUES cohort reported that having any economic insecurity was associated with worse PROs regardless of poverty, income, and SLE factors.^9^ Economic insecurities, conventional SDoH, and SLE severity vary across geographic regions in the United States.^30^ Therefore, the relationship between economic insecurities and PROs needs to be further elucidated in different SLE cohorts. Our findings are timely and important because they validate the negative impact of economic insecurities on PROs in a Midwestern cohort, enhancing the generalizability and reliability of these findings.

Illustrating the sequential burden of economic insecurities on PROs and clinical outcomes can clarify who could benefit from regular screening. Studies in other chronic diseases, such as diabetes, have shown that having multiple (more than two) economic insecurities was linked with a three‐fold higher risk of nonadherence and a five‐fold higher anxiety rate.^31^ Likewise, the CLUES cohort noted significantly lower PRO T scores across physical function, pain, fatigue, sleep, cognitive function, depression, and anxiety domains in patients with multiple economic insecurities.^9^ Consistent with these findings, we found that higher counts of economic insecurities (at least one, at least two, and at least three) were associated with progressively worse physical health T scores (−2.0, −3.9, and −9.1, respectively). Similarly, a significant linear association between increasing economic insecurities and lower PROs in all three domains was noted. Cumulatively, our findings support that patients with two or more economic insecurities are at higher risk of poor PROs and may benefit from routine screening for economic insecurities in clinics.

Additionally, quantifying interactions between economic insecurities and conventional SDoH can help explain the complexities driving heterogeneity in clinical outcomes and PROs in SLE. Our subgroup analysis revealed interactions between conventional SDoH and economic insecurities, informing the model shown in Figure 2. For example, having to rely on Medicaid or having no insurance combined with having economic insecurities led to a multiplicative negative impact on PROs, in which the observed PRO change was −4.38 compared to the expected additive change of −2.72 (−1.85, −0.87). This finding was consistent with our recent meta‐analysis highlighting a multiplicative negative impact of having to rely on Medicaid and experiencing care fragmentation or other economic barriers on clinical outcomes in LN.^8^ Likewise, a study reported that similar negative interactions among three or more adverse social factors drove a 1.26‐fold higher cancer mortality.^32^ However, such interactions were not seen in the CLUES cohort. This may reflect differences in Medicaid eligibility and coverage between California versus Wisconsin. Wisconsin has not fully expanded Medicaid, leaving adults in Wisconsin with incomes between 100% and 138% of the federal poverty level without coverage, potentially magnifying the impact of economic insecurities for the uninsured.^33^ These differences could explain contrasting interactions between Medicaid insurance and economic insecurities between the two cohorts.

This study provides new evidence on the use of a systematic workflow for SDoH screening and is the first to show that addressing economic insecurities in the clinics has a positive impact on PROs in SLE. To elaborate, both unadjusted and adjusted pre–post analyses showed significant improvements in physical health and social function domains after addressing economic insecurities. These findings are consistent with a systematic review that reported that having an SW in general medicine, pediatrics, or gynecology clinics had an overall positive effect on health outcomes and was less costly because it prevented hospital readmissions.^34^ These findings highlight that economic insecurities are modifiable risk factors and integrating routine screening as part of the SLE care plan could be beneficial. Second, the intervention developed for our Collaborative Lupus Clinics is generalizable, yet customizable, across different practices because it leverages a standard, validated EHR tool (Epic SDoH Snapshot) to screen for economic insecurities.^13^ Next, it provides workflows to integrate economic insecurities screening in clinics, even in busy practices. Finally, it provides guidance on how to integrate social support or community resources in SLE care plans to offer a comprehensive care plan and improve outcomes across all patients with SLE.

Despite strengths of our study, we do acknowledge limitations. First, data on other conventional SDoH such as poverty or literacy were not available for all patients because these data are not routinely reported in the EHR.^8^ Second, because this is a single‐center study from the Midwest, it may limit generalizability. Third, because our study leveraged the Epic EHR to assess economic insecurities, this may limit the corroboration of these findings in settings that use different EHR systems. However, the economic insecurities screening questions in Epic follow NAM and CMS guidelines, so similar screening across other institutions and EHRs is likely feasible, with some customization. This will be evaluated in future multicenter pragmatic studies. Finally, given limited sample sizes for patients with higher number of economic insecurities, we used cumulative categories to increase cell sizes for the higher exposure groups. Although this approach improved statistical power, and similar findings were noted with *P* for trend analysis, we acknowledge that larger longitudinal studies are needed to validate our findings.

To summarize, this study is unfolds key information on (1) modifiable high‐impact nonbiologic risk factors that should be screened in clinics (eg, transportation barriers, food insecurity, housing instability, financial insecurity) routinely, (2) a systematic method to integrate economic insecurities screening in clinics (eg, Epic SDoH Snapshot), and (3) the positive impact of partnering with an SW to provide a comprehensive care plan that addresses both biologic and nonbiologic factors (eg, SDoH, economic insecurities) on SLE outcomes. These findings lay a roadmap for clinical practice and future clinical research to integrate screening for nonbiologic factors as part of clinical care to advance health in chronic diseases such as SLE.

## AUTHOR CONTRIBUTIONS

All authors contributed to at least one of the following manuscript preparation roles: conceptualization AND/OR methodology, software, investigation, formal analysis, data curation, visualization, and validation AND drafting or reviewing/editing the final draft. As corresponding author, Dr Garg confirms that all authors have provided the final approval of the version to be published and takes responsibility for the affirmations regarding article submission (eg, not under consideration by another journal), the integrity of the data presented, and the statements regarding compliance with institutional review board/Declaration of Helsinki requirements.

## Supporting information


**Disclosure Form**:

**Supplementary Table 1.** Economic Insecurities Screening Questions from Epic SDoH Snapshot®.
**Supplementary Table 2.** Unadjusted Associations Between Economic Insecurities, Conventional SDoH, and PROs (n = 222).
**Supplementary Table 3.** Sequential Negative Impact of Increasing Economic Insecurities on Patient‐Reported: 1) Physical Health; 2) Mental Health; 3) Social Function (n=222).
**Supplementary Table 4.** Univariable Paired Pre‐Post Changes in PROs After Social Work Intervention Among Patients Reporting Economic Insecurities as Resolved at Follow‐Up (n=22).
**Supplementary Figure 1.** P for Trend Analysis Demonstrating Linear Associations Between Economic Insecurities and A) Physical Health, B) Mental Health, and C) Social Function.
