# Supplementary material for: Addressing Economic Insecurities Can Improve Patient‐Reported Outcomes in Lupus
Source: Arthritis Care Res (Hoboken). 2026 Mar 25;78(9):1177–87. doi: 10.1002/acr.80000 (PMC13511451; doi:10.1002/acr.80000)
Supplement: Supplementary file 2 [file ACR-78-1177-s002.docx]

**Supplementary Material for: “Addressing Economic Insecurities Can Improve Patient-Reported Outcomes in Lupus”**

Supplementary Table 1. Economic Insecurities Screening Questions from Epic SDoH Snapshot®

| **Economic Insecurity** | **Questions** |
| --- | --- |
| Housing Stability | - In the last 12 months, was there a time when you were not able to pay the mortgage or rent on time? *(Yes / No / Patient unable to answer / Patient declined)* - In the past 12 months, how many times have you moved where you were living? *(Free response)* - At any time in the past 12 months, were you homeless or living in a shelter (including now)? *(Yes / No / Patient unable to answer / Patient declined)* |
| Transportation Needs | - In the past 12 months, has lack of transportation kept you from medical appointments or from getting medications? *(Yes / No / Patient unable to answer / Patient declined)* - In the past 12 months, has lack of transportation kept you from meetings, work, or from getting things needed for daily living? *(Yes / No / Patient unable to answer / Patient declined)* |
| Food Insecurity | - Within the past 12 months, you worried that your food would run out before you got the money to buy more. *(Never true / Sometimes true / Often true / Patient unable to answer / Patient declined)* - Within the past 12 months, the food you bought just didn’t last and you didn’t have money to get more. *(Never true / Sometimes true / Often true / Patient unable to answer / Patient declined)* |
| Financial Resource Strain | - How hard is it for you to pay for the very basics like food, housing, medical care, and heating? *(Very hard / Hard / Somewhat hard / Not very hard / Not hard at all / Patient unable to answer / Patient declined)* - In the last 12 months, have you needed to see a doctor but could not because of the cost? *(Check box)* - In the last 12 months, did you skip medication to save money? *(Check box)* |

Footnotes: Questions used to screen for the presence of housing instability, transportation needs, food insecurity, and financial resource strain.

Supplementary Table 2. Unadjusted Associations Between Economic Insecurities, Conventional SDoH, and PROs (n = 222)

|  | **Physical Health** | | **Mental Health** | | **Social Function** | |
| --- | --- | --- | --- | --- | --- | --- |
| Variables at visit | Unadjusted Mean Change  (95% CIs) | p-value | Unadjusted Mean Change  (95% CIs) | p-value | Unadjusted  Mean Change  (95% CIs) | p-value |
| Age, per 10-Year Increase | 0.01  (-0.04, 0.07) | 0.63 | **0.08**  **(0.02, 0.14)** | **0.01** | 0.004  (-0.003, 0.01) | 0.21 |
| Female | 2.09  (-1.14, 5.32) | 0.20 | -0.71  (-4.14, 2.71) | 0.68 | -0.08  (-0.47, 0.31) | 0.69 |
| Black or African American | **-4.94**  **(-2.00, -7.89)** | **0.001** | **-5.51**  **(-2.40, -8.61)** | **0.001** | **-0.43**  **(-0.07, -0.79)** | **0.02** |
| Asian | 0.65  (-2.84, 4.15) | 0.71 | -0.78  (-4.47, 2.91) | 0.68 | -0.12  (-0.55, 0.30) | 0.56 |
| Hispanic or Latino | -0.91  (-5.22, 3.40) | 0.68 | -1.04  (-5.59, 3.51) | 0.65 | -0.18  (-0.70, 0.34) | 0.50 |
| Body Weight, per 1 kg Increase | **-0.06**  **(-0.01, -0.11)** | **0.009** | -0.02  (-0.07, 0.03) | 0.35 | -0.003  (-0.01, 0.002) | 0.27 |
| Medicaid Insurance/No Insurance | **-2.92**  **(-0.10, -5.74)** | **0.04** | **-3.54**  **(-0.56, -6.51)** | **0.02** | **-0.42**  **(-0.08, -0.76)** | **0.01** |
| Lupus Duration, per 1 Year Increase | -0.04  (-0.24, 0.16) | 0.70 | 0.05  (-0.16, 0.27) | 0.62 | 0.001  (-0.02, 0.03) | 0.94 |
| ADI <80% Decile, yes | -1.79  (-5.50, 1.91) | 0.34 | -1.73  (-5.65, 2.18) | 0.38 | -0.18  (-0.62, 0.27) | 0.44 |
| Loneliness or Depression | -1.30  (-5.37, 2.75) | 0.53 | -3.90  (-8.16, 0.37) | 0.07 | -0.26  (-0.75, 0.22) | 0.29 |
| High-Risk Health Behavior, per 1 Unit Increase | -0.51  (-5.57, 4.54) | 0.84 | 0.42  (-4.92, 5.76) | 0.88 | 0.12  (-0.48, 0.73) | 0.70 |
| Immunosuppression, yes | -0.80  (-2.07, 0.48) | 0.22 | -0.12  (-1.47, 1.23) | 0.86 | -0.05  (-0.20, 0.10) | 0.51 |
| Employment, no | **-4.26**  **( -1.81, -6.72)** | **0.001** | **-3.64**  **(-1.05, -6.23)** | **0.006** | **-0.62**  **(-0.33, -0.91)** | **0.00003** |
| Employment,  retired | -1.35  (-4.17, 1.48) | 0.35 | 2.42  (-0.56, 5.41) | 0.11 | 0.20  (-0.13, 0.53) | 0.23 |
| Employment, student | -0.14  (-5.39, 5.09) | 0.96 | 0.38  (-5.14, 5.91) | 0.89 | -0.01  (-0.70, 0.53) | 0.80 |
| SLEDAI, per 1 Unit Increase | -0.18  (-0.48, 0.12) | 0.23 | -0.13  (-0.44, 0.18) | 0.42 | -0.02  (-0.05, 0.02) | 0.38 |
| SDI, per 1 Unit Increase | **-1.22**  **( -0.36, -2.09)** | **0.01** | -0.46  (-1.39, 0.47) | 0.33 | -0.08  (-0.19, 0.02) | 0.13 |
| Prednisone Dose at Visit, per 1 mg Increase | **-0.12**  **(-0.01, -0.23)** | **0.04** | -0.06  (-0.18, 0.05) | 0.28 | **-0.02**  **(-0.002, -0.03)** | **0.02** |
| Economic Insecurities, per 1 Unit Increase | **-2.72**  **(-1.22, -4.23)** | **0.0004** | **-2.62**  **(-1.03, -4.22)** | **0.001** | **-0.34**  **(-0.17, -0.53)** | **0.0002** |
| HCQ Blood Levels >750 ng/ml, yes | -0.18  (-2.19, 1.84) | 0.86 | 1.06  (-1.06, 3.19) | 0.32 | 0.04  (-0.20, 0.28) | 0.72 |

Footnotes: Data are unadjusted mean change in PROs (95% CI, with p-value < 0.05 indicating significance). Lower scores indicate worse patient-reported outcomes. SDI = Systemic Lupus International Collaborating Clinics (SLICC) Damage Index. SLEDAI = Systemic Lupus Erythematosus Disease Activity Index. ADI = Area Deprivation Index.

Supplementary Table 3. Sequential Negative Impact of Increasing Economic Insecurities on Patient-Reported: 1) Physical Health; 2) Mental Health; 3) Social Function (n=222).

|  | **Economic Insecurities ≥1** | | **Economic Insecurities ≥2** | | **Economic Insecurities ≥3** | |
| --- | --- | --- | --- | --- | --- | --- |
| Patient Reported Outcome | Adjusted Mean Difference (95% CIs) | p-value | Adjusted Mean Difference (95% CIs) | p-value | Adjusted Mean Difference (95% CIs) | p-value |
| Physical Health | -2.00  (-5.20, 1.20) | 0.22 | -3.86  (-8.58, 0.87) | 0.11 | **-9.10**  **(-1.61, -16.6)** | **0.02** |
| Mental Health | -1.81  (-5.26, 1.65) | 0.30 | -2.59  (-7.71, 2.53) | 0.32 | -5.77  (-13.90, 2.39) | 0.17 |
| Social Function | -0.26  (-0.65, 0.13) | 0.18 | -0.44  (-1.01, 0.14) | 0.14 | **-1.33**  **(-0.42, -2.23)** | **0.004** |

Footnotes: Data are adjusted mean difference (95% CI, p value < 0.05 indicating significance) of PROS in patients with economic insecurity (≥1, ≥2, ≥3) compared to no economic insecurity. Lower scores indicate worse patient-reported outcomes.

Supplementary Table 4. Univariable Paired Pre-Post Changes in PROs After Social Work Intervention Among Patients Reporting Economic Insecurities as Resolved at Follow-Up (n=22)

| **Physical Health** | | **Mental Health** | | **Social Function** | |
| --- | --- | --- | --- | --- | --- |
| Unadjusted Mean Change  (95% CIs) | p-value | Unadjusted Mean Change  (95% CIs) | p-value | Unadjusted Mean Change  (95% CIs) | p-value |
| **5.25**  **(1.59, 8.91)** | **0.007** | 2.41  (-1.84, 6.66) | 0.25 | **0.65**  **(0.09, 1.21)** | **0.03** |

Footnotes: Data represent unadjusted mean changes in PROs from baseline to 3–6 month follow-up, shown with 95% confidence intervals (p < 0.05 indicating significance). Higher PRO scores indicate improved patient-reported outcomes.

Supplementary Figure 1. P for Trend Analysis Demonstrating Linear Associations Between Economic Insecurities and A) Physical Health, B) Mental Health, and C) Social Function.

A. Physical Health

B. Mental Health

C. Social Function
